# Commentary: Acute Effects of Exercise Mode on Arterial Stiffness and Wave Reflection in Healthy Young Adults: A Systematic Review and Meta-Analysis

**DOI:** 10.3389/fphys.2019.01516

**Published:** 2019-12-17

**Authors:** J. Derek Kingsley, Yu Lun Tai

**Affiliations:** ^1^Exercise Science/Physiology, Cardiovascular Dynamics Laboratory, Kent State University, Kent, OH, United States; ^2^Department of Health and Human Performance, The University of Texas Rio Grande Valley, Brownsville, TX, United States

**Keywords:** vascular, augmentation index, arterial stiffness, pulse wave velocity, opinion

## Introduction

We recently read the article, “Acute Effects of Exercise Mode on Arterial Stiffness and Wave Reflection in Healthy Young Adults: A Systematic Review and Meta-Analysis” by Doris R. Pierce, Kenji Doma and Anthony S. Leicht (volume 13, 2018). This particular article was a review and meta-analysis that focused on changes in arterial stiffness and measures of pulse wave reflection in response to different exercise modalities. The aim of this commentary is to highlight, and to make note, on some of the data that was presented in their manuscript.

Increases in measures of pulse wave reflection, primarily the augmentation index (AIx), and the AIx at normalized to 75 beats per minute (AIx@75), are associated with increased cardiovascular mortality and morbidity (Weber et al., 2004). The AIx is defined as augmentation pressure expressed as a percentage of aortic pulse pressure. It is influenced by the timing and the amplitude of the forward traveling wave and the reflected wave (Wilkinson et al., 2000). Following resistance exercise there appears to be a significant impact on measures of pulse wave reflection (Fahs et al., 2009; Yoon et al., 2010; Kingsley et al., 2017; Tai et al., 2018). This is further supported by the meta-analysis of Pierce et al. (2018).

## Concerns

However, while we were excited that our previous study, and data, are used in the meta-analysis by Pierce et al. (2018), we do have some concerns. Specifically, our primary concern revolves around the data taken from our article, Tai et al. (2018). In the article by Pierce et al., the data that are presented from Tai et al. (2018), specifically Figure 4, 1.1.2 Augmentation index, are incorrect. The data in reference are not the same in the article by Pierce et al. as they are in the article from Tai et al.

In our article, Tai et al., the AIx that was published was the peripheral AIx, and we did not correct it for changes in pulse pressure, the review article by Pierce et al. cited an AIx taken from Tai et al. that was not what was published. Tai et al. reported an augmentation index of 116.8 ± 4.2% at rest, and 123.2 ± 8.4%. However, Peirce et al. reported an augmentation index of 12.5 ± 6% at rest, and 19 ± 11.9% during recovery. From what we can tell, Peirce et al. calculated their own AIx, using our data and by dividing our reported AIx by augmentation pressure, but we are not certain. The correct data are such that resting AIx, corrected for pulse pressure, increased by 88.7%, while Peirce et al. reported that we had a 49.6% change.

## Discussion

Overall, our concern is that our data were re-calculated, incorrectly, and then used in the meta-analysis. We were not contacted about our data, and were most upset to see it presented in an incorrect manner. We feel that re-calculating the AIx from our data was not the best way to proceed. We don't know how much these incorrect data influenced the outcome of the meta-analysis.

## Author Contributions

All authors listed have made a substantial, direct and intellectual contribution to the work, and approved it for publication.

### Conflict of Interest

The authors declare that the research was conducted in the absence of any commercial or financial relationships that could be construed as a potential conflict of interest.
